# Supplementary material for: Mitochondrial Dynamics in Placenta-Derived Mesenchymal Stem Cells Regulate the Invasion Activity of Trophoblast
Source: Int J Mol Sci. 2020 Nov 14;21(22):8599. doi: 10.3390/ijms21228599 (PMC7696686; doi:10.3390/ijms21228599)
Supplement: Supplementary file 1 [file ijms-21-08599-s001.pdf]

Supplementary Materials

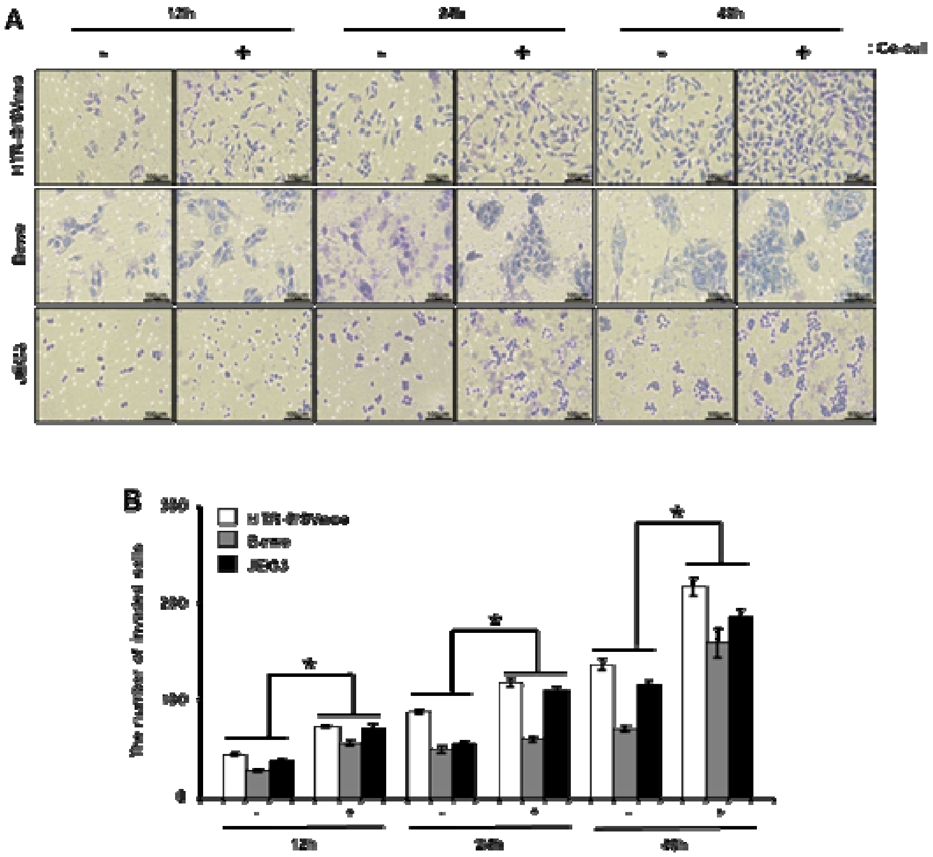

Supplementary Figure S1. PD-MSCs cocultivation induced invasion ability of different trophoblastic cell line. (**A**) Invasion ability of trophoblastic cell line was analyzed by trans well system. (**B**) The invaded cells were quantified by Image J program. The data was performed at least triplicate as the means ± S.D. Indicates p<0.05.

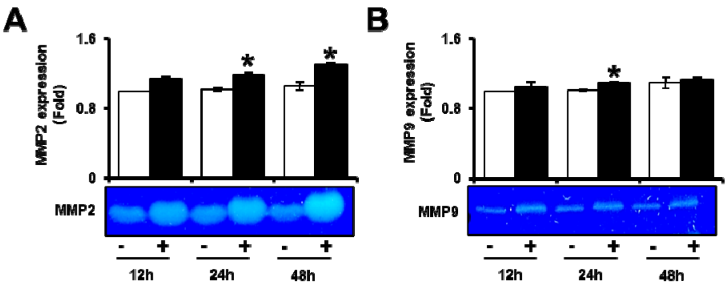

Supplementary Figure S2. PD-MSCs cocultivation induced MMP-2/-9 activities in trophoblast cells. (**A**) MMP-2 and (**B**) MMP-9 expression were analyzed by zymography. The intensity of MMP-2/-9 expression were quantified by Image J program. The data was performed at least triplicate as the means ± S.D. Indicates p<0.05.White bar: control, Black bar: PD-MSC cocultivation. *: control vs. PD-MSC cocultivation at each time point.

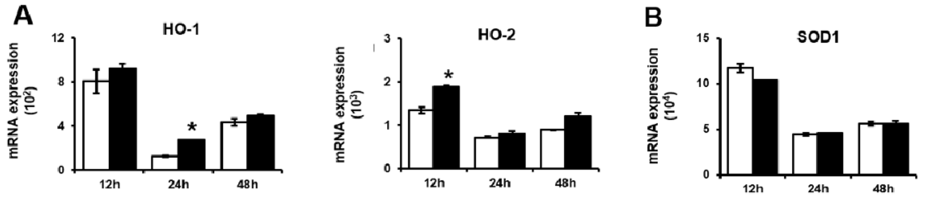

Supplementary Figure S3. PD-MSCs regulate oxidative stress in invasive trophoblasts. The mRNA expression of (a) HO-1/-2 and (b) SOD1 in invasive trophoblast with and without to PD-MSC cocultivation over time. The data was performed at least triplicate and expressed as the means ± S.D. Indicates $p<0.05$. White bar: control, Black bar: PD-MSC cocultivation. *: control vs. PD-MSC cocultivation at each time point.
